# High prevalence of headaches in patients with epilepsy

**DOI:** 10.1186/1129-2377-15-70

**Published:** 2014-11-04

**Authors:** Xiang-qing Wang, Sen-yang Lang, Mian-wang He, Xu Zhang, Fei Zhu, Wei Dai, Xiao-bing Shi, Min Wan, Yun-feng Ma, Ya-nan Chen, Sheng-yuan Yu

**Affiliations:** 1 Department of Neurology, Chinese PLA General Hospital, Fuxing Road 28, Haidian District, Beijing 100853, P.R. China

**Keywords:** Epilepsy, Headache, Migraine, Headache on seizure-day, Headache yesterday, Postictal headache

## Abstract

**Background:**

To examine the association between headaches and epilepsy.

**Methods:**

Consecutive adult epileptic patients who went to the outpatient clinic of the Epilepsy Center of PLA General Hospital between February 01, 2012, and May 10, 2013, were recruited into this study. A total of 1109 patients with epilepsy completed a questionnaire regarding headaches.

**Results:**

Overall, 60.1% of the patients (male: 57.2%; female: 63.8%) reported headaches within the last year. The age-weighted prevalence of interictal migraine was 11.7% (male 8.9%, female 15.3%), which is higher than that reported in a large population-based study (8.5%, male 5.4%, female 11.6%) using the same screening questions. The prevalence of postictal headaches was 34.1% (males 32.7%, females 35.2%), and the presence of preictal headaches was 4.5% (males 4.3%, females 5.2%). The prevalence of headache yesterday in the general population was 4.8% (male 3.0%, female 6.6%). Thus, the prevalence of headaches, including migraine, is higher in epileptic patients in China.

**Conclusions:**

The high prevalence of postictal headaches confirms the frequent triggering of a headache by a seizure. A much lower frequency of preictal headaches, a condition in which the real triggering effect of the headache on the seizure might be difficult to prove.

## Background

Primary headache disorders, particularly migraine and tension-type headache (TTH), are globally prevalent [1-5]. The prevalence of primary headaches is high in China and is not dissimilar to the world average, according to a previously population-based survey initiated by Lifting The Burden: the Global Campaign against Headache [6]. As an early project within the Global Campaign against Headache [7], an epidemiological study of headaches in China was initiated. This study used door-to-door calling to gather a random population-based sample drawn from all regions. The survey found a one-year prevalence of primary headache disorders in adults of 23.8% (males: 17.1%; females: 30.7%) [6], with a very substantial headache-attributed disability.

A population-based study of having a headache yesterday, which is almost free from recall bias, shows that in China, headaches affect 4.8% of adults aged 18–65 years on any given day [8]. Headaches cause approximately 1.3% of all days to be lost to disability (whether from usual daily activities, work or school). There are clear implications for health policy.

Epilepsy and headaches are the two most common neurologic disorders affecting individuals of all ages worldwide. There is a question of whether the high prevalence of headaches in patients with epilepsy in China is the same as in other regions. In a country with 1.3 billion people, the question is important, but there is very little evidence on which to base an answer.

The comorbidity between migraine and epilepsy has been well known for a century, but it is still not fully understood. The two disorders also share some symptoms, risk factors, and drug therapy. Several studies support the hypothesis of cortical excitability as a possible mechanism underlying their pathology, such as Na + −K + ATPase pump impairment, converging on a common final pathway represented by neuronal membrane hyperexcitability, could manifest as either epilepsy or headache/migraine [9]. Moreover, there is emerging evidence from both basic and clinical neurosciences that cortical spreading depression and an epileptic focus may facilitate each other [10].

An association between migraine and epilepsy has been demonstrated in several studies [11,12], but the data are complicated, and the studies have been limited by small numbers.

To date, no large-scale analysis has been conducted examining the association between headaches and epilepsy, especially in the Chinese population. The purpose of this study was to investigate the characteristics and prevalence of headaches in patients with epilepsy based on the International Classification of Headache Disorders, 2nd edition (ICHD-II) [13]. We aimed to compare our findings with those of a population-based epidemiological study of headache, which was a population-based door-to-door survey (PBDDS) [6,8].

## Methods

Consecutive adult patients with epilepsy, referred to the outpatient clinic of the Epilepsy Center of PLA General Hospital between February 01, 2012, and May 10, 2013, were recruited for this study. All the patients had a definite diagnosis of epilepsy as determined by at least two epileptologists. The patients included in this study were at least 18 years old and had the ability to autonomously answer the questionnaires. Patients with mental retardation, learning disabilities, behavioural disorders or other evident abnormalities that could compromise cooperation and the ability to respond the questionnaires were excluded. At the closing interview after one and a half years, 1109 patients, 607 men and 502 women, with a mean age of 28.2 (range 18–64) years, completed a questionnaire regarding headaches. All patients had performed the brain MRI examination, which showed normal in 231 (26.31%) patients. MRI showed 21.87% (192/878) of patients with mesial temporal scelerosis, 32.69% (287/878) with traumatic brain injury, 21.98% (193/878) with vascular (stroke, cavernomas, arteriovenous malformations), 8.20% (72/878) with lesions (tumors, focal cortical dysplasia), 9.57% (84/878) with inflammatory (encephalitis), and 4.56% (40/878) with others (encephalomalacia, or nonspecific MRI).

The diagnostic questions regarding headaches began with a screening question “Have you had a headache in the last year not related to flu, hangover, cold, head injury or other reasons?”, as recommended by earlier studies [14]. For the subjects who confirmed headaches, a standardized semistructured telephone interview was performed, with questions about headache timing in relation to seizures and about the frequency, duration, intensity, localization, and associated features of the headaches.

The headaches were further categorised as preictal, postictal or interictal. A preictal headache was defined as a headache starting not more than 24 h prior to the seizure and lasting until the onset of a seizure. A postictal headache was defined as a headache starting within three hours after a seizure and ceasing within 72 h after the attack. We further considered the headaches on seizure-day, which including the preictal and postictal headaches, and compared these results with the headache yesterday of the population [8].

Further questions regarding the headache on the seizure-day or the headache yesterday, including when it was reported and the duration (<1 hour, one to four hours, five to 12 hours or >12 hours, or lasting all day). The impact of the headache on usual daily activities (including work or school in those for whom yesterday was a work- or a school-day) was recorded according to the participants’ responses: unaffected (“could do everything as usual”), partially affected or totally affected (“could do nothing”). The patients were asked to grade their usual headache intensity as mild (maintaining normal activities without problems), moderate (maintaining normal activities with difficulty), severe (must give up normal activities and lie down) or extremely severe (impossible to stay still).

An interictal headache was defined as headache starting not earlier than three hours after a seizure or a headache never proceeding directly into an epileptic fit. Further diagnostic questions about the headache were based on the ICHD-II criteria [13] and aimed at identifying migraine and tension-type headache (TTH). The respondents who might have more than one type of headache were instructed to focus on the subjectively most bothersome type of headache. The questions included the frequency, duration, quality, site and intensity of headache, accompanying symptoms (nausea, vomiting, photophobia and phonophobia) and the impact of physical activity on the headache. To arrive at diagnoses from responses to these questions, the ICHD-II criteria were applied first for migraine, then for TTH, probable migraine, and finally for probable TTH. The remaining cases were considered unclassifiable. In calculating the prevalence of migraine, we added the cases of definite and probable migraine, as we did in the validation study (where the rationale is explained in detail) [14]. For TTH, we added the cases of definite and probable TTH.

Statistical analyses were performed with the Statistical Package for the Social Sciences (SPSS) version 14.0. The continuous variables were summarized as the means and standard deviations, and the categorical variables were summarized as numbers and percentages. Chi-square tests were used to compare the distributions of the categorical variables between the groups. Statistical significance was set at P <0.05. For this report, we validated the sample by comparing it, for age and gender distribution, with national statistics derived from the 2010 census for the population aged 18–65 years [6] and a general-population survey of the headache yesterday [8].

### Ethics

The study protocol was approved by the Chinese Ministry of Health and the ethics committee of the Chinese PLA General Hospital, Beijing. The participants provided their “written informed consent” when they were recruited for this study.

## Results

### One-year prevalence of headaches in patients with epilepsy

**Figure 1:**
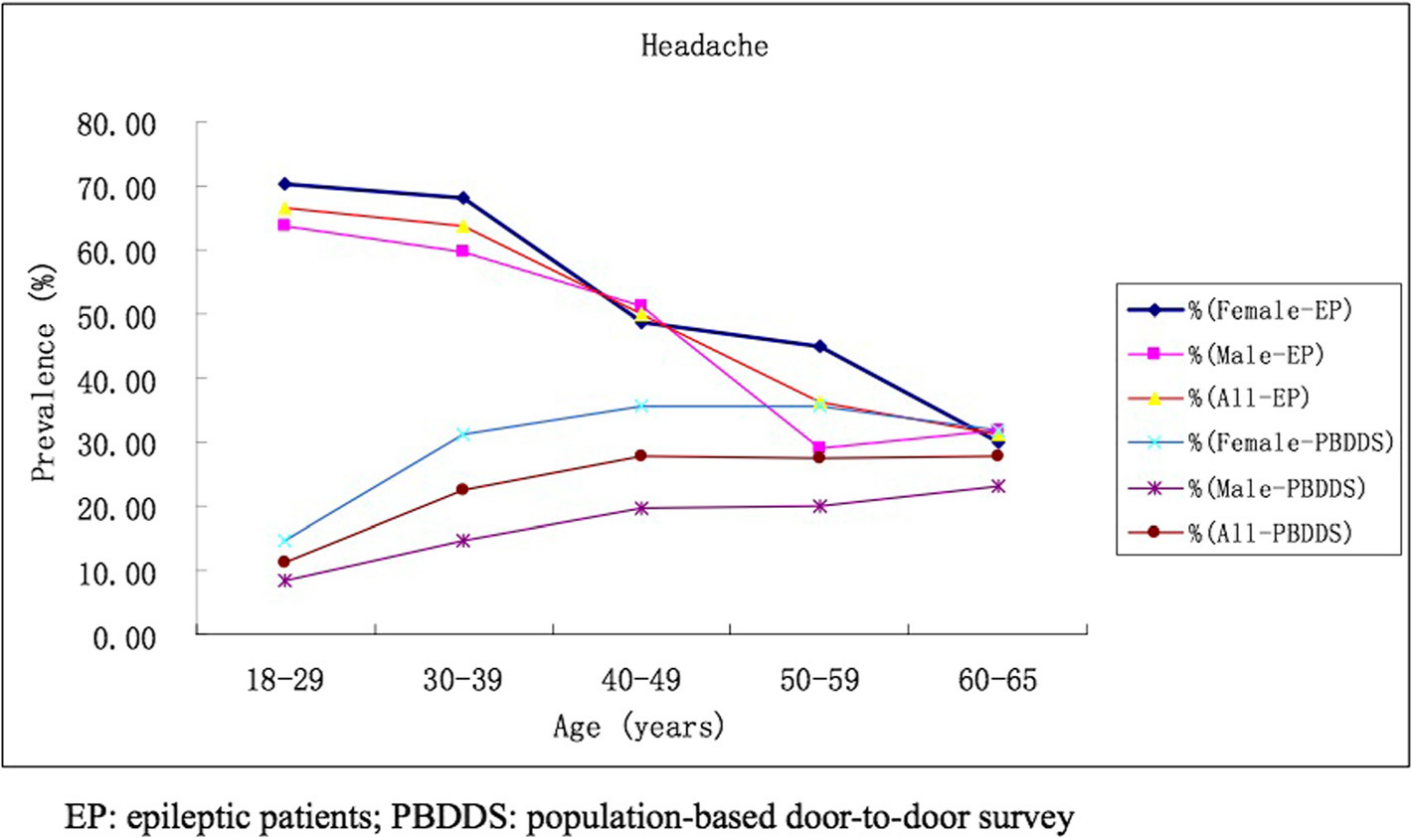
Prevalence of headache in patients with epilepsy compared to population-based door-to-door survey of headache in China.

In total, 1109 consecutive adult epileptic patients were included in this study, of which 607 (54.7%) were males and 502 (45.3%) were females. Overall, 60.1% (667/1109) of the patients reported headaches in the last year. Headaches were less prevalent in the males (57.2%) than in the females (63.8%) (p = 0.026). The age-weighted prevalence of headaches in epilepsy was 54.3% (male 51.6%, female 57.3%), which was higher than the previously results published a population-based door-to-door survey (PBDDS), which found a one-year headache prevalence of 28.4% in women and 15.6% in men (p <0.001) (6). There were no significant differences regarding the type of epileptic syndrome, the aetiology of epilepsy or MRI abnormalities between the patients with and without headaches. (Table 1) The prevalence of headaches in epileptic patients peaked during young adulthood (18–29 years), compared with middle age (40–49 years) in the population (Figure 1).

**Table 1:** Prevalence of headache in patients with epilepsy compared to a population-based door-to-door survey of headache in China

**Age (years)**	**Patients with epilepsy**		**Population-based survey (6)**		**p**	
	**Females**	**Males**	**Females**	**Males**	**Females**	**Males**
18-29	182 (70.27%)	209 (63.72%)	61 (14.7%)	36 (8.3%)	0.0000	0.0000
30-39	81 (68.07%)	71 (59.66%)	157 (31.4%)	83 (14.6%)	0.0000	0.0000
40-49	36 (48.65%)	46 (51.11%)	236 (35.7%)	127 (19.7%)	0.0287	0.0000
50-59	18 (45.0%)	14 (29.17%)	196 (35.5%)	116 (19.9%)	0.2275	0.1253
60-65	3 (30%)	7 (31.82%)	112 (31.8%)	76 (23.1%)	0.9031	0.3604

### Preictal headaches

Fifty-nine patients had headaches that evolved into seizures, and the headaches were categorised as migraine for 38 of these patients. In 48 patients, the headache started within 30 min prior to the seizure, and 11 patients had headache 24 h to 30 min before seizure onset. We found three patients had an attack of migraine with aura followed immediately by a seizure, which included two occipital lobe epilepsy (OLE) cases (one caused by trauma and one caused by meningitis) and one case of secondary epilepsy with Systemic lupus erythematosus. The visual aura lasted approximately 10–20 minutes. In two OLE cases a migraine-like aura and a migraine itself could be the first phase on an occipital epileptic seizure, therefore preceding the other epileptic manifestations. The following is the detail of the two occipital lobe epilepsy cases.

Case1: 62-year-old female with a history of traumatic brain injury in the left occipital lobe (about thirty years ago), complained of episodic rapid flashing or flickering achromatic lights in the right side, lasting 1–3 minutes, then headache for about 30 minutes. She will sometimes stare and lose awareness for about 2-5minutes. Attacks have been occurring 3–7 times per year, good response to the drug (phenytoin, PHT). Her MRI was abnormal which showed a softened lesion in the left occipital lobe, interictal EEG confirmed the left occipital spkies.

Case 2: 33-year-old male with a history of viral meningoencephalitis about two months ago, complained of visual hallucination for 1–3 minutes, bad headache about 10 minutes, then will make a sound and his both arms and legs will make movements, being a generalized tonic-clonic seizure. Seizures have been occurring daily. His brain MRI was normal, interictal EEG showed asymmetrical bilateral posterior sharp-slow wave complexes. He was administrated with adjunctive therapy (VPA 500 mg bid, and LTG 50 mg bid), discharge from hospital 20 days later with a good control of seizure.

### Prevalence of interictal headaches

**Figure 2:**
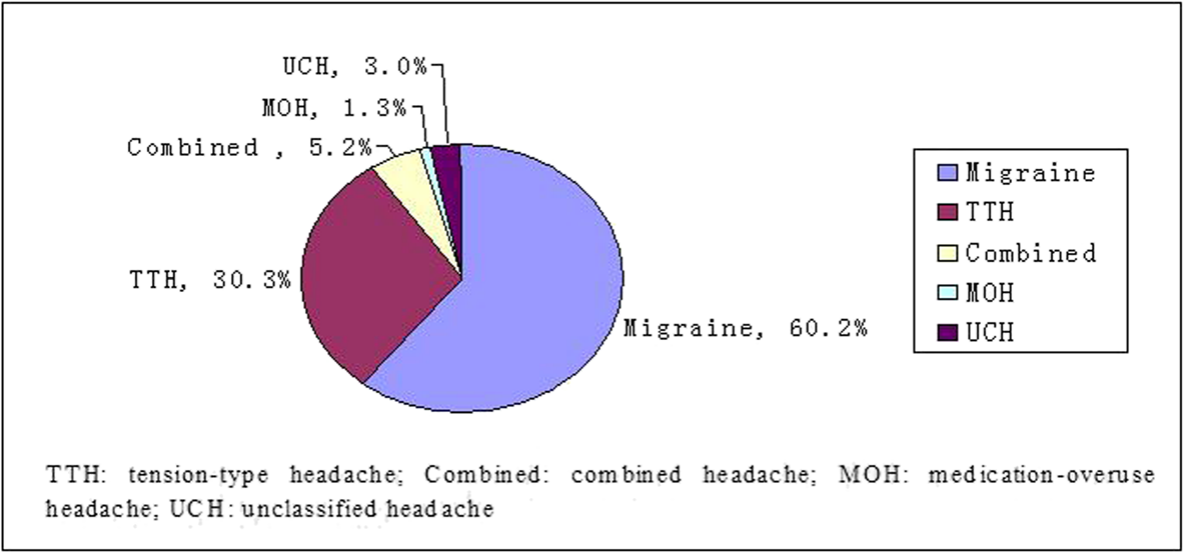
Diagram of types of interitcal headache in patients with epilepsy (n = 231).

Among the 231 epileptic patients with this type of headache (including 54 patients who had postictal headaches and interictal migraine and 17 patients who had postictal headaches and interictal tension-type headaches), the pain was mild for 72 (31.2%) patients, moderate for 131 (56.7%) patients and severe for 28 (12.1%) patients. Migraine headaches (including probable migraine) were present in 60.2% of the patients with interictal headaches: 30.3% had tension-type headaches, 5.2% had combined headaches and 4.3% had other headache types (Figure 2).

The prevalence of interictal migraine, TTH, combined headache, medication-overuse headache, and unclassified headache in our epileptic patients was 139/1109 (12.5%), 70/1109 (6.3%), 12/1109 (1.1%), 3/1109 (0.3%), 7/1109 (0.6%), respectively. The age-weighted prevalence of interictal migraine was 11.7% (male 8.9%, female 15.3%), which is higher than was reported in a large population-based study (8.5%, male 5.4%, female 11.6%) using the same screening questions.

**Figure 3:**
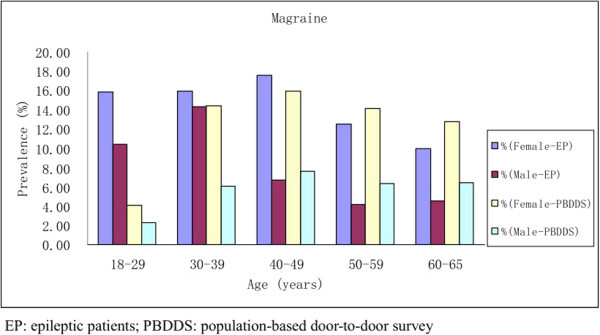
Prevalence of interictal migraine in patients with epilepsy compared to a population-based door-to-door survey of headache in China.

The prevalence of interictal migraine in female patients peaked during middle age (40–49 years), which is in contrast to the 30–39 year-old peak in the male patients (Table 2 and Figure 3). Females (15.7%, 79/502) were nearly twice as likely to suffer from interictal migraine as males were (9.9%, 60/607). The prevalence of interictal migraine in epilepsy was significantly higher than the population-based study in subjects aged 18–29 years and 30–39 years (p <0.001); there were no differences in the subjects aged greater than 40 years between the two groups. (Table 2 and Figure 3).

**Table 2:** The prevalence of interictal migraines in patients with epilepsy compared with a population-based door-to-door survey of headaches in China

**Age (years)**	**Patients with epilepsy**		**Population-based survey (6)**		**p**	
	**Females**	**Males**	**Females**	**Males**	**Females**	**Males**
18-29	41 (15.8%)	34 (10.4%)	17 (4.1%)	10 (2.3%)	0.0000	0.0000
30-39	19 (16.0%)	17 (14.3%)	72 (14.4%)	35 (6.1%)	0.0003	0.0022
40-49	13 (17.6%)	6 (6.7%)	105 (15.9%)	49 (7.6%)	0.7085	0.7562
50-59	5 (12.5%)	2 (4.2%)	78 (14.1%)	37 (6.3%)	0.7743	0.7482
60-65	1 (10.0%)	1 (4.6%)	45 (12.8%)	21 (6.4%)	0.7943	0.9183

### Prevalence of headache on seizure-day and headache yesterday

There were 498 patients with epilepsy who reported headaches on the seizure-day, and the age-weighted prevalence of headaches on the seizure-day was 38.6% (male 37.0%, female 40.5%), including 439 patients with postictal headaches, 59 patients with preictal headaches and 18 patients with both preictal and postictal headaches. The prevalence of postictal headaches was 34.1% (males 32.7%, females 35.2%), while that of preictal headaches was 4.5% (males 4.3%, females 5.2%). Of the 498 patients, the headache on the seizure-day lasted all day in 132 (26.5%), for <1 hour in 118 (23.7%), between 1 and 4 hours in 89 (17.9%), between 5 and 12 hours in 130 (26.1%) and longer than 12 hours in 32 (6.4%). Among the 498 patients who reported the intensity, the headache on the seizure-day was mild in 164 (32.9%), moderate in 267 (53.6%) and severe in 67 (13.5%) (Table 3).

**Table 3:** The prevalence, duration, intensity and impact of a headache on the seizure-day and headache yesterday by gender

	**Patients with epilepsy**		**Population-based survey (8)**	
	**Females**	**Males**	**Females**	**Males**
Prevalence of this type of headache (n/N of participants %)				
	146 (56.4%)	174 (53.1%)	195/2480 (7.9%)	91/2561 (3.6%)
Duration of headache (%)				
<1 hour	46/235 (19.6%)	72/263 (27.4%)	25/181 (13.8%)	13/85 (15.3%)
1-4 hours	48/235 (20.4%)	41/263 (15.6%)	62/181 (34.3%)	33/85 (38.8%)
5-12 hours	59/235 (25.1%)	71/263 (27.0%)	25/181 (13.8%)	8/85 (9.4%)
>12 hours	18/235 (7.7%)	11/263 (4.2%)	1/181 (0.6%)	1/85 (1.2%)
All day	64/235 (27.2%)	68/263 (25.9%)	68/181 (37.6%)	30/85 (35.3%)
Intensity of headache (%)				
Mild	73/235 (31.1%)	91/263 (34.6%)	38/182 (20.9%)	16/86 (18.6%)
Moderate	122/235 (51.9%)	145/263 (55.1%)	115/182 (63.2%)	58/86 (67.4%)
Severe	40/235 (17.0%)	27/263 (10.3%)	29/182 (15.9%)	12/86 (14.0%)
Impact on daily life, work or school of headache (%)				
Unaffected	82/235 (34.9%)	103/263 (39.2%)	58/183 (31.7%)	28/86 (32.6%)
Partially affected	127/235 (54.0%)	143/263 (54.4%)	101/183 (55.2%)	46/86 (53.5%)
Could do nothing	26/235 (11.1%)	17/263 (6.5%)	24/183 (13.1%)	12/86 (14.0%)
Took medication for headache (%)				
	4/235 (1.7%)	2/263 (0.8%)	124/178 (69.7%)	64/85 (75.3%)

**Figure 4:**
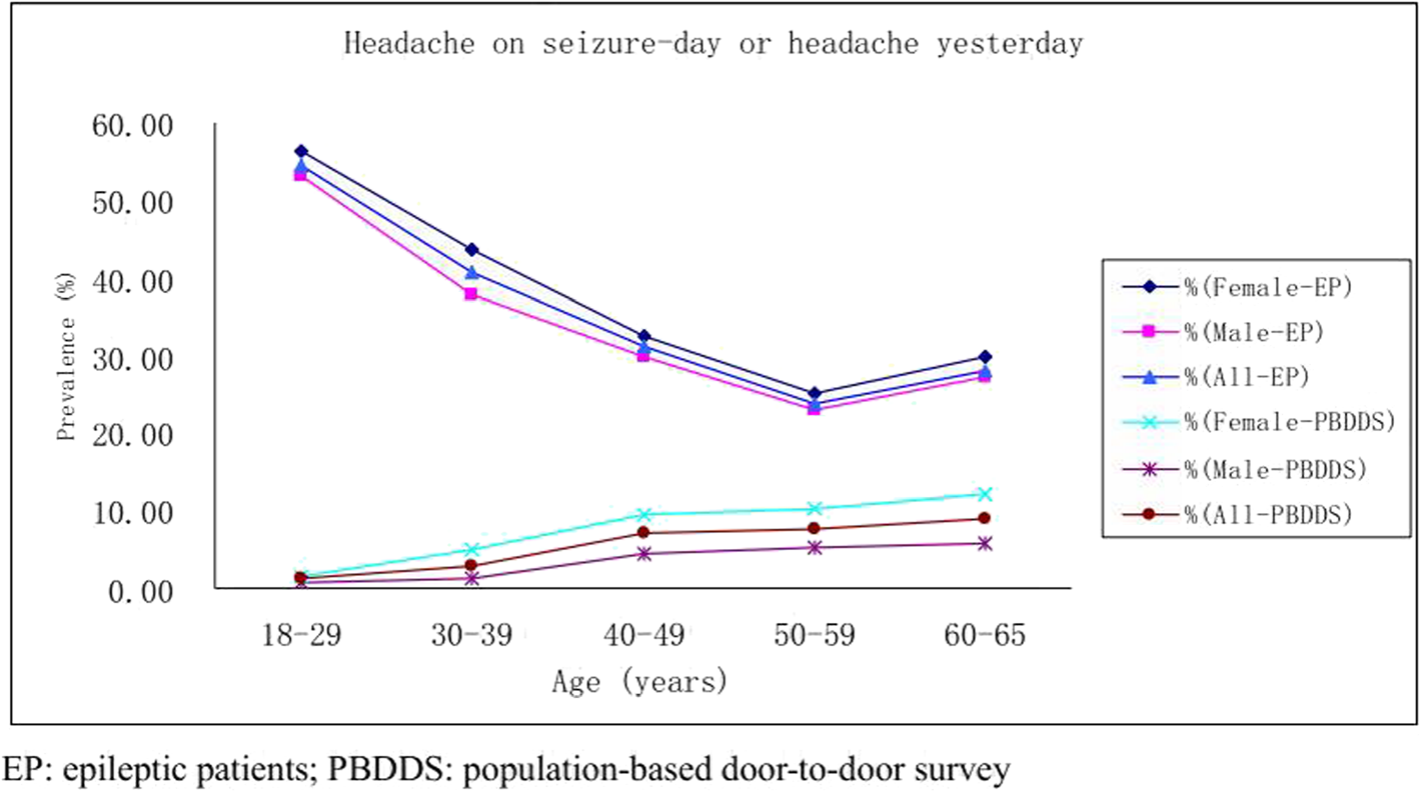
Prevalence of headache on seizure-day in epileptic patients and headache yesterday a population-based door-to-door survey of headache in China.

Across China, 286 (5.7%) (male 3.6%, female 7.9%) of the 5041 participants reported a headache yesterday (8), and the age-weighted prevalence was 4.76% (male 3.0%, female 6.6%). (Table 4 and Figure 4) Headaches on the seizure-day are most common in young patients (18–29 years), and the prevalence declined with age throughout the range of 18–59 years. Comparisons of the age distributions in the population with a headache yesterday shows, skewing toward the older age groups, with the prevalence increasing with age throughout the range of 18–65 years. (Table 4 and Figure 4).

**Table 4:** The prevalence of a headache on the seizure-day compared with headache yesterday in a population-based door-to-door survey of headaches in China

**Age (years)**	**Patients with epilepsy**		**Population-based survey (8)**		**p**	
	**Females**	**Males**	**Females**	**Males**	**Females**	**Males**
18-29	146 (56.4%)	174 (53.1%)	7 (1.7%)	4 (0.9%)	0.0000	0.0000
30-39	52 (43.7%)	45 (37.8%)	25 (5.0%)	7 (1.2%)	0.0000	0.0000
40-49	24 (32.4%)	27 (30.0%)	63 (9.5%)	30 (4.6%)	0.0000	0.0000
50-59	10 (25.0%)	11 (22.9%)	57 (10.3%)	31 (5.3%)	0.0047	0.0015
60-65	3 (30.0%)	6 (27.3%)	43 (12.2%)	19 (5.8%)	0.0959	0.0002

## Discussion

The prevalence of headaches is high in patients with epilepsy (57.2%) in China and is not dissimilar from the world average. Headaches were less prevalent in males (51.6%) than in females (57.3%). In the population-based study, the prevalence of headaches peaked during middle age (40–49 years) in contrast to young adulthood (18–29 years) in the epilepsy group. These epidemiological results were first reported and stressed by Verrotti et al. [15-17], which conducted in pediatric age. Moreover, Belcastro V. et al. [18] have reported that the epidemiological behavior in this field is clearly different comparing adult and pediatric patients. Our findings also confirm that headaches are considerably more common in people with epilepsy compared with the average population, and the prevalence may have the relationship with age.

Our previous reports [19] have shown that there was no significant difference regarding type of epileptic syndrome, and the frequency of seizures between patients with headaches and without headaches, which was similar with a study from Korea Headache in Epileptic Patients Study Group [20], they also did not found a significant relationship between seizure type, and the intensity or frequency (in association with seizure) of the seizure-related headache (SRH). Their results also showed that there was no significant difference in the frequency of overall SRH or postictal SRH between patients with GTCS (GTCS in generalized epilepsy, or seizure types including secondarily GTCS in partial epilepsy) and patients without.

We have investigated the incidence of postictal headache (PIH) and the factors potentially related to the occurrence of PIH [21], which showed that PIH occurs more frequently after generalized tonic–clonic seizures than other seizure types. Our findings are consistent with Ito et al. [22], and Botha SS et al. [23] who suggested that PIH occurs commonly in generalized.

The one-year prevalence of interictal migraine in our epileptic patients was higher than the large population-based door-to-door survey of headaches in China. The prevalence of headaches is related to gender and age. The one-year prevalence of headaches and migraine in epileptic patients and in the general population are higher in women than in men in China. Females were nearly twice as likely to suffer from interictal migraine as were males. In the epilepsy group, the prevalence of interictal migraine in female patients peaked during middle age (40–49 years), compared with a peak in males aged 30–39 years. In the population survey, the prevalence of migraine increased with age until a peak was reached during the fifth decade of life; thereafter, the prevalence declined, more quickly in women than in men. These findings are similar to other studies [24-27]. The fact that migraine is the most common in young to middle aged adults may demonstrate that the prevalence of headaches in the epilepsy group peaked during a younger age period than in the population survey.

Headache yesterday is a new concept in headache epidemiology, which to avoid recall bias inherent in inquiries covering prolonged periods (typically three months) in the past. The purpose of comparing "headaches on day of seizure" in the epileptic group to "headaches yesterday" in the general population was, also, to focus on burden. Patients and doctors often pay more attention to epileptic attack rather than the epileptic-related headache. We asked the patients to recall headaches occurring on the seizure-day, which could be remembered clearly by the patients. Then compare these two groups, which are all headaches in just one day and all could be recalled clearly, to show the exactly different burden and relationship between the two common disorders. Our findings suggest that headaches on the seizure-day are considerably more common in the epilepsy group compared with the headache yesterday in the average population. Headaches on the seizure-day are most common in young patients (18–29 years), and the prevalence declines with age throughout the range of 18–59 years. A comparison of the age distributions in the population with headache yesterday shows skewing toward the older age groups, increasing with age throughout the range of 18–65 years. Headache yesterday was more than twice as likely in women than in men (7.9% vs 3.6%). Men and women had similar results with regard to the headache duration and intensity and the measures of functional impairment. The difference reflects, and is explained by, the higher one-year prevalence of headaches in women in China [6], as has been shown elsewhere. The high prevalence of postictal headaches confirms the frequent triggering of a headache by a seizure [28]. Schon and Blau [29] showed that all the patients with postictal headaches have at least one characteristic of migraine, such as vomiting, photophobia, or phonophobia. The authors suggest that, as has been postulated for migraine, postictal headaches might be related to the vasodilatation known to follow seizures. Conclusive pathophysiological concepts of postictal headaches cannot yet be proposed.

There was a much lower frequency of a preictal headaches, a condition in which the real triggering effect of the headache on the seizure may be difficult to prove [30].

The association between epilepsy and headache (especially migraine) is particularly interesting, as there are all common medical disorder that has multiple phenotypes with complex and poorly understood underlying mechanisms. Recent data have even reported that familial hemiplegic migraine (FHM) and benign familial infantile convulsions (BFIC) can be associated with the same novel genetic mutation in the Na + −K + ATPase pump [9]. Na + −K + ATPase pump defects, due to cortical excitability alterations, can result in phenotypic manifestations of both migraine and epilepsy, which are due to cortical spreading depression (CSD) (for migraine) and inhibitory postsynaptic potential (IPSP) (for epilepsy) modulations, respectively. Badawy et al. [31] used transcranial magnetic stimulation (TMS) to assess cortical excitability in migraine compared with control subjects and patients with epilepsy. They found that cortical excitability increases in migraine suggesting the involvement of intracortical inhibitory circuits. This may be a common feature underlying some of the similarities observed in migraine and epilepsy. Although there was a matter of debate about whether seizures or CSD causes true migraine typical, CSD seems to be the connecting point between migraine and epilepsy [32].

Migralepsy is an old term deriving from migra(ine) and (epi)lepsy that has been used for the first time by Lennox and Lennox to describe a condition in which "ophthalmic migraine with perhaps nausea and vomiting was followed by symptoms characteristic of epilepsy". However, the concept of migralepsy as a migraine-epilepsy sequence is too narrow and inadequate. The term "ictal epileptic headache" has been recently proposed to classify the clinical picture in which headache is the isolated ictal symptom of a seizure [33]. There is emerging evidence from both basic and clinical neurosciences that cortical spreading depression and an epileptic focus may facilitate each other, although with a different degree of efficiency. So the new term “ictal epileptic headache” concept and the published criteria have been recently mentioned in the “appendix” of the new edition (third edition) of the ICHD-3, published in Cephalalgia. It should be keeping in the mind that headache or visual symptoms can be the epileptic “aura” of a seizure, as it has been shown in the case description of a patient with a partial status epilepticus in occipital lobe epilepsy [34].

Most people with headaches in China do not seek medical care. Similar findings have been reported by others [35,36]. The common beneficial effect of over-the-counter analgesics may explain why this issue is rarely discussed with a doctor. Moreover, the patients hesitate to treat their headaches for fear of having to take more drugs, and their doctors do not ask them about headaches and therefore do not advise them of appropriate treatment. People with epilepsy should be encouraged to discuss their headaches with their doctor, and the doctors should routinely ask patients with epilepsy about headaches.

### Reliability of the study

**Figure 5:**
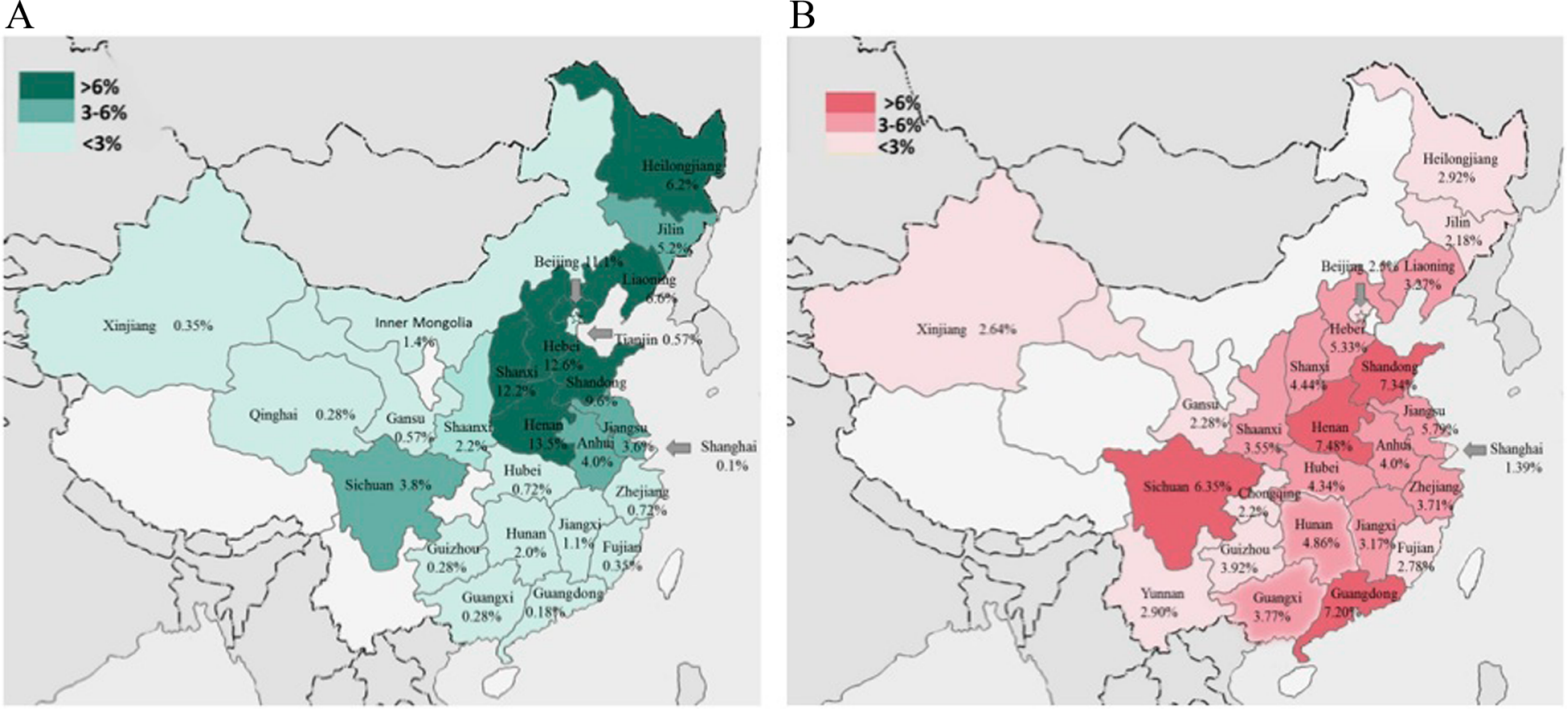
**Regional distribution of epileptic patients in the current study (n = 1109) and people in a population-based door-to-door survey of headache in China (n = 5041). A**: Epileptic patients came from 25 regions of China. Colors from dark to light indicate different proportion of epileptic patients from higher to lower (3 levels: >6%, 3-6%, and <3%). **B**: People in epidemiological study of headache came from 25 regions of China. Colors from dark to light indicate different proportion from higher to lower (3 levels: >6%, 3-6%, and <3%).

This is a large-scale cross-sectional study to investigate the one-year prevalence of headaches in patients with epilepsy in China and to compare the results with those of a population-based epidemiological study of headaches and headache yesterday, a population-based door-to-door survey. A major limitation of this study is a possible recall bias, although the questionnaire about headaches on the seizure day had been designed to avoid this bias, the accuracy of some data may not be yet good enough. We hope to perform the prospective investigation about headache and epilepsy to avoid this bias in the future study.Another limitation of this study is the differences in the selection of patient populations; the patients with epilepsy come from the outpatient clinic of the Epilepsy Center of PLA General Hospital, but the population-based door-to-door survey was based on random-sampling of people from 22 provinces and 3 directly administered cities. Our institution is one of the largest hospitals in China, which includes the patients from 21 provinces and 4 directly administered cities (Figure 5). This result may reflect the real relationship between epilepsy and headaches in China.

A shortcoming of this study is that this is a questionnaire-based investigation about the prevalence of headache in patients with epilepsy, and the accuracy of some data may be poor. We did not include an EEG recording of the peri-ictal phase, so we cannot “demonstrate or rule out" if peri-ictal cases could be an "epileptic headache" (cases where headache can be the sole ictal epileptic manifestation) [37].

We asked the patients to recall headaches occurring on the seizure-day, and we asked them to differentiate preictal from postictal headaches. The diagnosis of a postictal headache is a headache starting within three hours after a seizure and ceasing within 72 h after the attack. We considered a headache with a duration lasting over 24 h an all-day headache which should compared with headache yesterday in the general population. This could more directly show the relationship between epilepsy and headaches.

## Conclusions

Our results indicate that headaches are more common in patients with epilepsy. The prevalence of migraine in our epileptic patients was higher than in the large population-based study. We asked the patients to recall headaches occurring on seizure-day, and we asked them to differentiate preictal from postictal headaches. The high prevalence of post-ictal headaches confirms the frequent triggering of a headache by a seizure. There was a much lower frequency of a preictal headaches, which are a condition in which the real triggering effect of the headache on the seizure might be difficult to prove.

This comorbidity is important because headaches often receive less attention than the more acute and dramatic symptoms of seizures. In epilepsy, questions concerning migraine should be an integral part of the history because the comorbidity might influence the antiepileptic drug choice, and the migraine might require specific treatment. This insufficient attention adds significantly to the burden of epilepsy, but the nature of the underlying pathogenesis remains to be clarified.

## Competing interests

All authors declare there are non-financial competing interests (political, personal, religious, ideological, academic, intellectual, commercial or any other) in relation to this manuscript.

## Authors’ contributions

MD, WXQ, LSY, XMW, ZX, ZF, DW, SXB, WM, MYF, CYN and YSY carried out the studies. And WXQ drafted the manuscript. M.D. WXQ participated in the design of the study and performed the statistical analysis. Professor YSY, the PI of this study, conceived of the study and participated in its design and helped to draft the manuscript. All authors read and approved the final manuscript.
